# Adsorption dynamics of O_2_ on Cu(111): a supersonic molecular beam study†

**DOI:** 10.1039/d3cp01215h

**Published:** 2023-05-17

**Authors:** Diyu Zhang, Charlotte Jansen, Aart W. Kleyn, Ludo B. F. Juurlink

**Affiliations:** a Leiden Institute of Chemistry, Leiden University PO Box 9502 2300 RA Leiden The Netherlands l.juurlink@chem.leidenuniv.nl (+31) 71 527 4221

## Abstract

We have studied the adsorption of O_2_ on Cu(111) using supersonic molecular beam techniques. For incident energies ranging between 100 and 400 meV, we have determined the sticking probability as a function of angle of incidence, surface temperature, and coverage. Initial sticking probabilities range from near 0 to 0.85 with an onset near 100 meV, making Cu(111) considerably less reactive than Cu(110) and Cu(100). Normal energy scaling applies and reactivity increases appreciably over the entire range of surface temperatures from 90 to 670 K. A strictly linearly decreasing coverage dependence on sticking precludes adsorption and dissociation *via* an extrinsic or long-lived mobile precursor state. We cannot exclude that sticking also occurs molecularly at the lowest surface temperatures. However, all tell tales from our experiments suggest that sticking is predominantly direct and dissociative. Comparison to earlier data shows implications for the relative reactivity of Cu(111) *vs.* Cu/Ru(0001) overlayers.

## Abstract 2

Schematic diagram of the O_2_ molecular collide onto the intrinsic sites and extrinsic sites.

## Graphical abstract

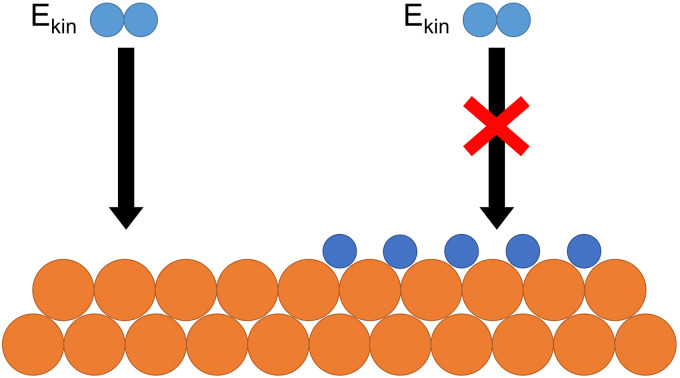

## 1 Introduction

The adsorption of O_2_ and formation of atomic O overlayers on metal surfaces are initial steps in many wanted and unwanted chemical processes, such as electrocatalysis, heterogeneous catalysis, and corrosion.^1–5^ As a cheap active metal, copper is widely applied as a catalyst in the synthesis of methanol.^6,7^ Consequently, the interaction of O_2_ with different Cu surfaces has attracted large research interest and has been examined *via* both theoretical and experimental studies.^2^ Experimental techniques that have been applied in combination with single crystal surfaces to control the surface structure are supersonic molecular beam techniques, low energy electron diffraction (LEED), scanning tunneling microscopy (STM), Auger Electron Spectroscopy (AES), X-ray photoelectron spectroscopy (XPS), electron energy loss spectroscopy (EELS) and reflection absorption infrared spectroscopy (RAIRS), among others.^8–14^

Supersonic molecular beam techniques are powerful tools to investigate the dynamics and kinetics of gas–solid surface reactions.^15^ They allow for control over the kinetic energy and the relative directions of motion of the reactants. As the reactivity is probed under single collision conditions and energies of the gas and solid reactants are separately controlled, direct *vs.* indirect reaction mechanisms can be identified, *e.g.* based on the influence of surface temperature and collision angle on the reactivity.

Nesbitt and coworkers applied such techniques in the study of O_2_ adsorption on Cu(110). They studied the dependence of the initial sticking probability (*S*_0_) on kinetic energy (*E*_kin_) and surface temperature (*T*_surf_).^16,17^ The dependencies showed that the dissociative chemisorption of O_2_ on Cu(110) proceeds *via* two channels. Activated, direct dissociation *via* an early barrier occurs as well as trapping-mediated dissociative adsorption.^17^ Anisotropy in the potential that affects adsorption and dissociation was studied in great detail using space-quantization and alignment of the impinging O_2_ molecules by Kurahashi and coworkers.^18^ For Cu(100), Valden and coworkers interpreted the adsorption dynamics of O_2_ also as including a precursor or steering-mediated adsorption at defects.^19^ Kasai and co-workers suggest different roles of vibration and rotation of O_2_ in the dissociation process for all low Miller index Cu surfaces, but did not address whether Cu(111) also participates in precursor and direct dissociation processes in parallel.^9^

A recent theoretical study suggests that O_2_ sticks mostly dissociatively to Cu(111) under single collision conditions.^20^ The process occurs *via* a molecular state in which the O_2_ molecule may be trapped if the incident kinetic energy barely exceeds the entrance channel activation barrier to molecular adsorption. Using a RPBE-XT potential energy surface (PES), a minimum energy barrier for entrance channel activated molecular adsorption was found to be 97 meV for the top-bridge-top (t-b-t) orientation with the O_2_ internuclear axis parallel to the surface. At this site, dissociation cannot occur. However, a minor displacement of the O_2_ molecule parallel to the surface plane toward the bridge-fcc-bridge (b-fcc-b) site opened up barrier-free dissociation. Kinetic energy-dependent initial sticking probabilities were computed with quasi-classical trajectory (QCT) calculations for this PES. Application of a Generalized Langevin Oscillator (GLO) modeled surface temperature effects and molecule–surface energy exchange, and has taken energy dissipation to the bulk into account. These calculations suggested an exponential increase in *S*_0_ with *E*_kin_ with values increasing from ∼1 × 10^−2^ to 0.7 over a kinetic energy range from 100 to 200 meV. At higher energies, *S*_0_ decreases slightly. While at 350 K, sticking was found to be nearly exclusively dissociative, at 100 K sticking was found to be mostly molecular. Comparison to very limited experimental data for the energy dependence to sticking^21^ indicated that general trends were captured, but quantitative agreement was lacking. The experimental data, obtained previously to compare the reactivity of Cu(111) to Ru overlayers on the same surface, suggested much lower reactivity of the clean Cu(111) surface than that of the Ru-covered surface.

In a recent co-adsorption study of CO on partially oxidized Cu(111), we used background dosing of O_2_ instead of molecular beam techniques.^12^ When using background dosing, molecules impinge on the surface with a kinetic energy distribution governed by the temperature of the vacuum chamber's wall, *i.e.* room temperature. The angle of impact is also randomized. We interpreted the results of surface oxidation by CO titration and RAIRS detection of the CO internal stretch frequency to indicate that only surface defects act as a source of O_2_ dissociation under these conditions. Rather large O_2_ doses are required to oxidize Cu(111) to any significant extent and the internal stretch absorbance of post-dosed CO was shown to diminish nearly linearly in intensity with an increased O_2_ dose, but without any effect on the internal stretch frequency. In line with previous studies, we argued that the results suggest that Cu(111) oxidation from background dosed O_2_ initiates at step defects and creates stoichiometric Cu_2_O patches that grow terrace-inward. STM studies confirm this mechanism for collision energies associated with dosing O_2_ as a bulb gas at room temperature.^11^

In this paper, we study the chemisorption of O_2_ on Cu(111) in more detail. Contrary to our previous study, where we had no control over the incident O_2_ molecules that impact on the surface, we now apply supersonic molecular beam techniques. We determine the sticking probability (*S*) as a function of incident energy and angle of incidence, surface coverage, and surface temperature, *S*(*E*_kin,⊥_, *θ*, and *T*_surf_).

## 2 Experimental

Experiments are performed in a double differentially pumped supersonic molecular beam system with a base pressure of approximately 1 × 10^−10^ mbar in the sample chamber. The details of this home built apparatus have been described before.^22,23^ Briefly, the apparatus contains, among others, an Auger electron spectrometer (AES, ESA100, Staib Instruments), a fixed quadrupole mass spectrometer (QMS, Pfeiffer, QMA200) for King and Wells (KW) measurements, and a second QMS (Pfeiffer, QMA125) that can be moved continuously along the molecular beam axis for time of flight measurements.

The Cu(111) single crystal (6N, Surface Preparation Laboratory, Zaandam, the Netherlands) is mounted at the bottom of a liquid nitrogen cooled cryostat on an *x*, *y*, *z*, *θ* manipulator. A K-type thermocouple is welded on the single crystal to measure the sample temperature. The Cu(111) surface is cleaned by Ar ion sputtering (5 × 10^−6^ mbar Ar, 10 mins, *T*_surf_ = 400 K) and followed by annealing (in vacuum, 10 mins, and *T*_surf_ = 800 K) This procedure is repeated for at least 3 cycles prior to every measurement of the sticking probability. The surface cleanliness was confirmed by AES. The same Cu crystal and cleaning procedures were applied in our recent study of O_2_ adsorption by background dosing as part of a CO/O coadsorption study with a different UHV system.^12^

The molecular beam is generated by expansion of a gas mixture at approximately 5 bar through a tungsten nozzle with a 28 μm diameter orifice. Gas mixtures are created using two mass flow controllers (Bronkhorst, the Netherlands) that each controls the flow of a single high purity gas. The mixture is created by both entering a long stainless steel line which feeds the nozzle and expansion. Each mixture is created and expanded continuously for at least 30 minutes to ensure the stability of the gas mixture prior to any measurements. The molecular beam is shaped by two molecular beam skimmers and a final circular orifice. It can be modulated by two flags and a variable-speed wheel chopper. The kinetic energy of the molecular beam is controlled by seeding O_2_ (N5.8, Airproducts) in helium (N6, Linde). The kinetic energy of the O_2_ in the molecular beam is determined by the time of flight (TOF) experiments with the movable QMS as described in the ESI.† The sticking probability is obtained by KW measurements.^24^ We find that the effective pumping speed in the UHV chamber is affected when cooling the crystal's cryostat with liquid nitrogen (LN2). It affects the shape of our KW traces for experiments performed at cryogenic crystal temperatures. In the ESI,† we describe how we handle possible influences on *S*_0_ and *S*(*θ*).

## 3 Results

**Fig. 1:**
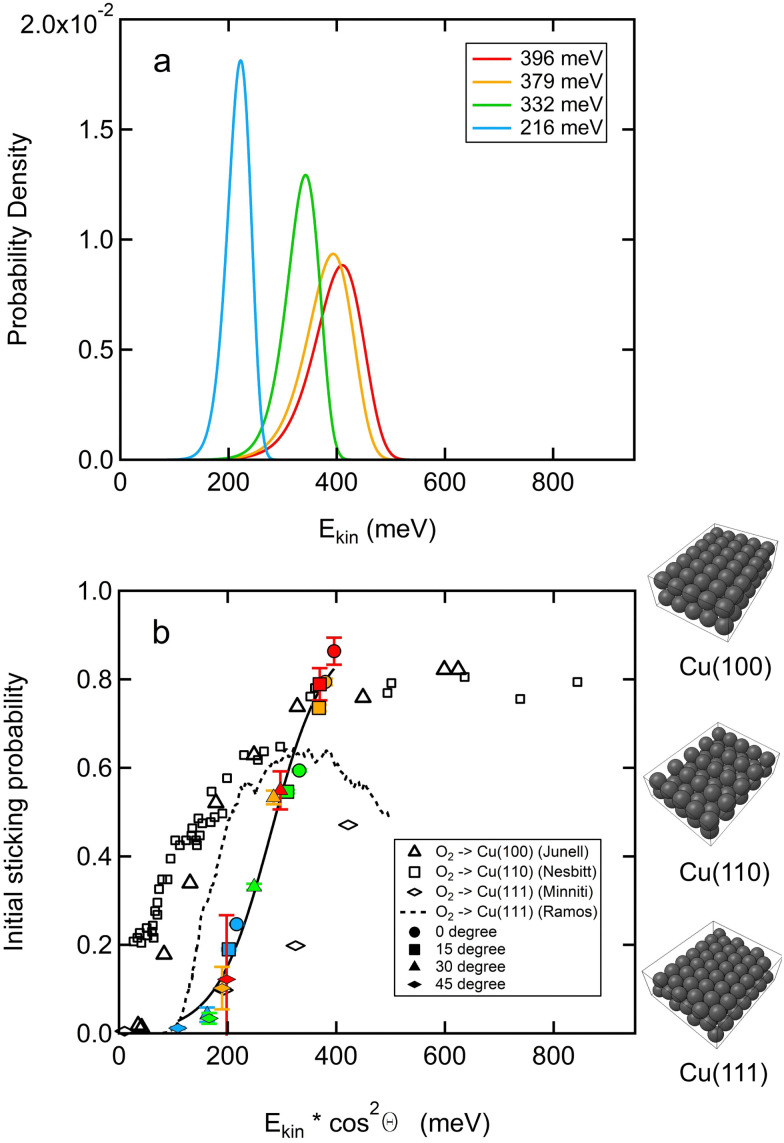
(a) Kinetic energy distribution of various O_2_ supersonic molecular beams used in this study. Incident energies are indicated in the legend. (b) *S*_0_ at *T*_surf_ = 300 K as a function of average normal incident kinetic energy (filled markers). The color coding reflects the different molecular beam energy distributions in a. Incident angles are indicated in the legend. Error bars reflect the uncertainty from the time-dependent fit for KW measurements as described in Fig. S1 (ESI†). The experimental data for *S*_0_ as a function of average normal incident kinetic energy is fitted with the model given by Harris (solid black line).^25^ Previous experimental data for Cu(111) (open diamonds)^21^ and simulation (black dashed line).^20^ Also shown: experimental data for Cu(100) (open squares)^19^ and Cu(110) (open triangles).^17^

Fig. 1a shows the kinetic energy distribution of the O_2_ supersonic molecular beams, which have been used to collect sticking probability data in this study. The energy distributions are calculated from time-of-flight measurements where we vary the neutral flight path length of O_2_ in the molecular beam. Time-dependent signals are corrected for the sensitivity of electron-ionization based detection and converted using the appropriate Jacobians to velocity and energy distributions.^26^ From the energy distribution, we calculate the average kinetic energy and use this to plot data as in Fig. 1b.

Fig. 1b shows the dependence of *S*_0_ for O_2_ sticking on Cu(111) as a function of kinetic energy at *T*_surf_ = 300 K, but scaled for normal incidence. At this surface temperature, sticking is solely dissociative. The kinetic energy of the O_2_ molecular beam is varied by changing the incident angle and the seeding ratio of the molecular beam. The average kinetic energy of O_2_ for different seeding ratios is listed in Table S1 (ESI†). The solid markers of a single color were measured with the molecular beam's energy distribution indicated with the same color in Fig. 1a. The shape of the marker represents the incident angle as indicated in the legend. The nozzle temperature was fixed at room temperature to exclude variations of rotational or vibrational contributions to dissociation.^9^ We also show previously published experimental data for Cu(111) (open diamonds),^21^ andCu(110) and Cu(100) (open squares and triangles),^17,19^ and the results of the recent theoretical study (black dashed line).^20^ Our results show that the normal component of the incident energy contributes to the adsorption process, while the parallel component of the incident energy is completely inefficient. This behavior is typical for activated adsorption. *S*_0_ also follows the typical S-shape for activated adsorption and increases from approximately 0 to 0.85 over a kinetic energy window ranging from approximately 100 to 400 meV. This indicates a narrow activation barrier distribution.

The experimental data are fitted with the following formula:^25^1
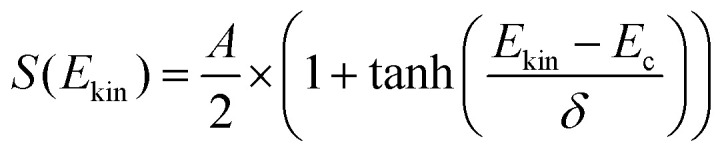
where *E*_kin_ is the normal incident kinetic energy, *E*_c_ is the critical energy (the transitional energy at which the sticking probability is half of the maximum) and *δ* is the width of the function. Note that a potential contribution of vibrational energy to the critical energy is ignored here, because the molecular beam is set at room temperature at which most O_2_ molecules are at the ground vibration state. A contribution to *S*_0_ by the small population of excited oxygen is negligible. The value of *E*_c_ is found to be 279 meV and *δ* equals 100 meV. We note that our fit is of a rather simple form and better approaches for extracting characteristic values of barrier distributions have been applied to H_2_/Cu(111).^27^

Our experimental data qualitatively reflect the results of the theoretical simulation (black dashed line in Fig. 1b). The calculated minimum entrance-channel activation barrier for O_2_ sticking on Cu(111) of 97 meV^20^ agrees nicely with the onset of dissociation as determined by the KW limit for measuring sticking (∼1 × 10^−2^). The previous experimental study by Minitti *et al.* (open diamonds in Fig. 1b) does not specify an onset for dissociation.^21^ Interpolation of their data to extract an onset is also difficult to justify for reason of the low number of data. Except for the value at 200 meV, *S*_0_ is significantly smaller in their results. Alternatively, one could argue that their sticking probabilities are shifted to higher *E*_kin_. This may be caused by differences in the determination of the kinetic energy of the various seeded beams. As Minitti *et al.* obtain their beam energy by the use of an empirical relationship and not by experimental determination using time-of-flight, quantitative comparison is difficult. However, when we use the same relationship as used by Minitti *et al.* to calculate the expected kinetic energies for our various beams, we obtain very similar values compared to the TOF analysis of our beams. Our data would, thus, not shift significantly if we use their approach. In addition, their *S*_0_ curve of O_2_ on clean Ru(0001) is almost identical to the one published previously by Wheeler *et al.*^28^ Another potential reason for a discrepancy is that our sticking probabilities appear higher due to the smaller width of the energy distributions present in our molecular beams. Although this may be the case, we do not expect such a large effect. We use comparable expansion conditions and previously found quantitative agreement when studying dissociation of H_2_ on Ru(0001).^29^ We conclude that there seems to be a clear difference between the data from the two different experiments with an unidentified cause. We speculate that it relates to the cleanliness and history of the Cu(111) surface.

Summarizing, our results in Fig. 1b clearly reflect an activation barrier to dissociation of O_2_ on Cu(111) which agrees with theoretical simulations. The initial sticking probability begins to increase at around 100 meV and reaches 0.85 at 379 meV with no suggestion of a drop in reactivity for higher incident energies. In this incident energy range, the initial sticking probability scales with the normal component of the incident energy. In comparison to Cu(100) and Cu(110), the significant activation barrier on Cu(111) makes it much less reactive.

**Fig. 2:**
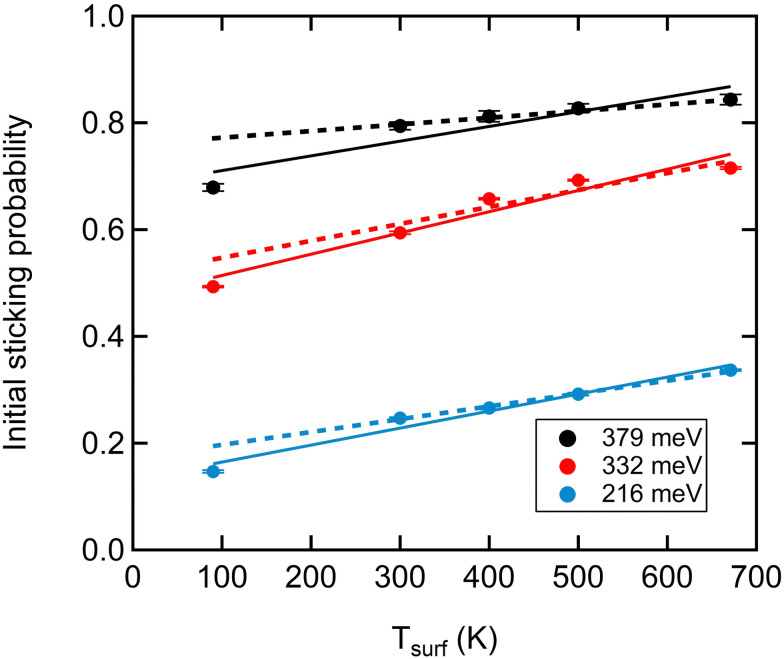
*S*_0_ *versus* surface temperature for three incident kinetic energies as indicated in the legend (see text for details). Error bars reflect the uncertainty from the time-dependent fit for KW measurements as described in Fig. S1 (ESI†).

Fig. 2 illustrates the effect of the surface temperature on *S*_0_ for averaged normal incident beam energies of 379 meV, 332 meV and 216 meV, respectively. The data have been fitted with two sets of linear functions. When we take all data for a single kinetic energy into account, we find the solid lines. When we exclude the data for *T*_surf_ = 90 K, we obtain the dashed fits. The comparison shows that in both cases, *S*_0_ clearly increases with an increase in the surface temperature. However, the slope is in all cases significantly smaller when excluding the data obtained at 90 K (2.8 *vs.* 1.3, 3.9 *vs.* 3.1, and 3.2 *vs.* 2.5 × 10^−4^*K*^−1^ for the fits applied to increasing *E*_kin_). Hence, extrapolation of data obtained from 300 to 670 K and at 90 K consistently leads to an overestimate of *S*_0_ of approximately 0.05 at 90 K.

**Fig. 3:**
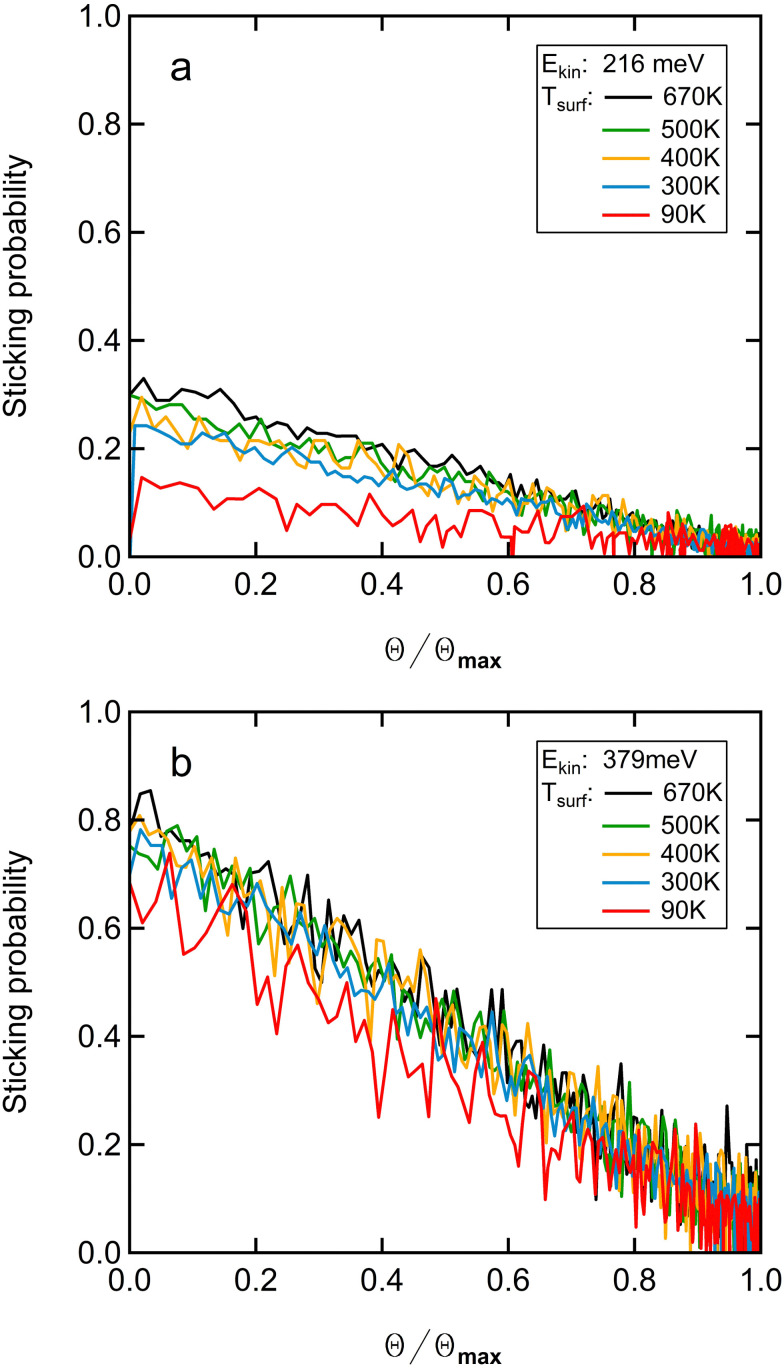
The sticking of O_2_ on Cu(111) as a function of *θ*/*θ*_max_ at 216 meV (a) and 379 meV (b) normal incidence energy for *T*_surf_ = 90 K (red), 300 K (blue), 400 K (yellow), 500 K (green), 670 K (black). *S*(*θ*) as a function of *θ*/*θ*_max_ at 379 meV (normal incidence) for 90 K < *T*_surf_ <670 K.

Fig. 3 shows the sticking probability as a function of fractional coverage, *θ*/*θ*_max_, at 216 meV (top panel) and 379 meV (bottom panel) of normal incidence energy for surface temperatures ranging from 90 to 670 K. The fractional coverage is obtained by integrating the uptake of O_2_ in KW traces. The uptake is then normalized to the maximum integral for each single measurement. As our QMS channeltron's amplification is dependent on the history of experiments performed with oxidating and reducing gases, we can, unfortunately, not compare absolute signals or integrals thereof.^30^ Consequently, we can also not establish whether the maximum obtainable coverage of *O*_ads_ (*θ*_max_) is kinetic energy dependent, as was shown for, *e.g.*, CH_3_ resulting from direct CH_4_ dissociation on Pt(111)^31^ and N_2_ dissociation on W(110).^32^ We can only establish the relationship between S and *θ*/*θ*_max_ for each incident energy. The results in both panels of Fig. 3 are, however, clear in this respect. We find rather strictly linear dependencies across the entire surface temperature range for both incident energies. In the ESI,† we show that there is no evidence for curvature by analyzing the residuals of the signal and the linear fits.

## 4 Discussion

For the interpretation of our results in terms of adsorption and dissociation mechanisms, we need to distinguish between supersonic molecular studies and all other studies of the interaction of Cu(111) with background dosed O_2_. Recent studies of O_2_ dissociation as achieved by background dosing agree that dissociation on Cu(111) is dominated by defect sites. The STM study by Lawton *et al.*^11^ reports dissociative chemisorption on a large range of Cu surfaces present at a dome-shaped Cu crystal (d-Cu(111)-10° in the description by Auras and Juurlink^33^. Oxidation leads to the formation of stoichiometric Cu_2_O at room temperature with reactivity (initially) being proportional to step density. The defect type was shown to be irrelevant as both straight and kinked steps show identical results. Our own RAIRS-based study of CO post-adsorbed to partially oxidized Cu(111) also indicated that background dosing leads to initial oxidation of defect sites.^12^ Stoichiometric Cu_2_O patches initiate at (step) defects and grow similar to oxidation of other noble metal surfaces, *e.g.* Ag(111).^34,35^ The growth of the oxide patches reduces remaining clean Cu(111) surface area that binds post-dosed CO. The CO is otherwise unaffected as evidenced from the exact same internal stretch frequency.

The defect-dominated oxidation mechanism obtained by background dosing is entirely different from the previous and present results of molecular beam studies and we must, therefore, be careful in all comparisons with earlier studies of the interaction of O_2_ with Cu(111) and other low-Miller index surfaces. In particular, we need to be wary of conclusions drawn from studies such as those from HREELS measurements of O_2_ dosed onto Cu(111) that claim both atomic and molecular states at low surface temperatures.^14^ Molecular states appearing at larger doses (on the order of 10^1^–10^3^ L) do not necessarily reflect O_2_ binding to pristine Cu(111) and may be affected by the initial and relatively facile oxidation of naturally occurring (step) defects.

Focusing on the present data and comparing to data from earlier molecular beam studies, we find in Fig. 1b that *S*_0_ for Cu(111) is significantly lower than for Cu(100) and Cu(110) at all normal incidence energies up to approximately 400 meV. As the normal incidence energy decreases, *S*_0_ on Cu(110) decreases but stabilizes at 0.2. Previous studies associate such probability behavior with precursor-mediated adsorption^36^ or steering-mediated adsorption.^37^ For Cu(111), we find that the initial sticking probability continues to drop toward 0 with decreasing normal incident kinetic energy. At normal kinetic energies above 100 meV, dissociation is substantial and well above the residual defect density that may be expected for a cleaned and annealed Cu(111) surface, *i.e.* ≪1%. With the strong and positive energy-dependence of *S*_0_ reported in Fig. 1b, it is clear that molecular beam experiments primarily detect the dissociation on defect-free parts of the Cu(111) surface. Hence, while precursor-mediated adsorption and (step) defects on Cu(111) or the stepped structure of Cu(110) may dominate dissociative adsorption for O_2_ molecules with kinetic energies typical for room temperature bulb gasses, this seems not the case for Cu(111) at energies used here.

The temperature-dependence reported in Fig. 2 is also in contrast to long-lived and mobile precursor-mediated adsorption. According to the standard precursor-mediated adsorption model, increasing the surface temperature would lower the sticking probability as this reduces the precursor lifetime, hence favoring desorption over diffusion that would be followed by dissociation. The initial sticking probability of O_2_ on Cu(111), however, increases as the surface temperature increases (Fig. 2). This opposite behavior was previously explained by the so-called recoil effect. Increased motion of surface atoms effectively lowers the required kinetic energy for the impinging molecules to pass the transition state, as discussed by Hand and Harris for H_2_ direct dissociative adsorption on Cu.^38^ The argument was also invoked to explain the dissociative chemisorption of O_2_ on Cu(100).^39^ The combination of increasing *S*_0_ with both surface temperature and normal kinetic energy over the entire probed range only supports direct activated dissociative adsorption on pristine Cu(111) sites.

Our interpretation of results presented in Fig. 1 and 2 call into question earlier suggestions of a relevance of a molecular state, as reported in experimental work^14^ and the recent theoretical DFT-based and dynamics study.^20^ Tsuyoshi *et al.* reported a molecularly adsorbed oxygen state on Cu(111) with electron energy loss spectroscopy when the surface temperature was below 230 K.^14^ Only after annealing up to 230 K, the molecularly adsorbed oxygen was argued to dissociate forming atomic oxygen. The recent DFT-based study also finds a clear molecular state that binds O_2_ with more than 100 meV. The binding energy varies slightly within the surface plane. On the bridge site with the internuclear axis pointing toward the top of neighboring Cu atoms (t-b-t), binding is slightly less strong compared to a laterally shifted position over a threefold hollow site (b-fcc-b). When considering the dynamics, one would expect that if a precursor state contributes to trapping and subsequent dissociation, its contribution may be substantial at low incident energies and surface temperatures, as also suggested by the dynamical calculations on the PES.^20^ The contribution would fade with increasing energy and temperature as direct dissociation starts dominating. Although it seems reasonable that a precursor-mediated contribution adds to (or perhaps dominates over) direct dissociation under low energy conditions, the extrapolation of our surface temperature dependent data in Fig. 2 contrasts it. The reactivity at 90 K is consistently lower than that expected from the extrapolation to 90 K from higher surface temperatures where dissociation must occur. The difference of roughly 0.05 is significantly larger than our expected error in these KW measurements. Had there been additive molecular and dissociative processes available, one would have expected *S*_0_ at 90 K to be higher than the extrapolations – not measurably lower.

We may also consider the coverage dependence of sticking and dissociation. Fig. 3 shows that it is linear for all temperatures up until *S*(*θ*) reaches zero at *θ*_max_. This behavior contradicts dissociative chemisorption *via* an extrinsic precursor, in which the O_2_ adsorbs first on (O-covered) Cu(111) and by diffusion finds an empty site for dissociation. If precursor mediated adsorption occurs on the (oxidized) surface, the sticking probability would be nearly constant up to an intermediate coverage and then drop quickly at higher coverages.

For more consideration of a potential contribution of an intrinsic precursor, we realize that a linear dependence of the sticking probability on coverage only agrees with a functional form

2 *S*(*θ*) = *S*_0_ × (1 − *θ*/*θ*_max_)^*N*^

for *N* = 1.^32^ A value of *N* = 2, as could be argued on the basis of Langmuir-type O_2_ dissociation and requiring two adsorption sites for the final state of two *O*_ads_ atoms, would yield clear curvature at both the start and end of the sticking probability curve. The lack thereof suggests that dissociation proceeds *via* a single type of site. This could be the molecular state found in the PES indicated as the b-fcc-b site that leads to non-activated dissociation from a weakly bound molecular state. However, as argued earlier, the observed temperature dependence of *S*_0_ is contradictory to a long-lived precursor. Also, the surface temperature dependence fitted to higher surface temperatures and extrapolated to 90 K yields an overestimate of the actual sticking probability. Hence, we conclude that if dissociation indeed takes place *via* a molecular state on Cu(111), that this is only a transient (and likely not equilibrated) state over the kinetic energy range probed here. Such a dynamic precursor state that includes minor diffusion with the unit cell has been suggested for H_2_ dissociation on step sites of Pt(111),^22,40^ and for which absolute reaction cross-sections have been determined.^41^ For O_2_ dissociative adsorption on Cu(110), charge transfer causing creation of a short-lived intermediate *O*_2_^*δ*−^ state on the way to dissociation at higher impact energies was suggested from a combined theoretical and experimental study that involved state-selection and alignment of impacting O_2_.^18^ For the current system, we believe that a similar intermediate species may be involved, although the main bottle neck to dissociation on defect-free Cu(111) seems to be the barrier in the entrance channel. According to the PES, this barrier varies at least between approximately 100 and 200 meV for impact with the internuclear axis parallel to the surface. Once this barrier is overcome, dissociation is facile and – depending on the exact impact site – may involve some steered motion parallel to the surface. The geometric corrugation for the molecular state seems very large from the reported cuts of the PES.^20^ Molecular scattering experiments at higher incident energies, spin and alignment control of the impacting and scattering O_2_,^18,42^ or angle- and energy-resolved scattering/desorption measurements^43^ may provide evidence.

Finally, we reiterate that in an absolute sense, we find mostly higher reactivities for O_2_ impinging onto Cu(111) than the only earlier published results.^21^ Whereas at 200 meV incident energy the data match rather well, at higher incident energies our values of *S*_0_ are considerably closer to what Minitti *et al.* report for Cu overlayers on Ru(0001). This calls into question the size of the enhanced reactivity claimed for these overlayers of Cu/Ru(0001) in comparison to Cu(111). This also impacts the reported changes in reactivity for Cu overlayers on Ru in comparison to pure Cu. While enhanced reactivity of the overlayers of Cu/Ru(0001) as compared to clean Cu(111) is still significant near 200 meV, it has vanished around 400 meV.

## 5 Conclusion

We have studied the sticking probability of O_2_ on Cu(111) as a function of coverage, surface temperature, and normal incidence transition energy, and compared the results to previous experimental and theoretical results. The dissociative chemisorption of O_2_ on Cu(111) bears all characteristics of a direct, activated process with a minimum barrier of approximately 100 meV. While at higher surface temperatures, sticking is surely dissociative, at the lowest surface temperature probed here (90 K), we believe dissociation also dominates, although we cannot exclude a contribution of molecular sticking in a precursor state. The temperature dependence to initial sticking may reflect a recoil effect that lowers the entrance channel activation barriers. It may also affect the efficiency with which motion parallel to the surface is induced and which is suggested to be required for following the minimum energy path to dissociation. Finally, we note that molecular states as found in earlier experiments that used background gas dosing of O_2_ likely need to be readdressed for reason of the now understood mechanism by which thermal O_2_ oxidizes the Cu(111) *via* defect sites.

## Author contributions

DZ performed nearly all experiments with the help of CJ. DZ prepared the manuscript. AK and LJ guided in the experiments and interpretation and took part in finalizing the manuscript.

## Conflicts of interest

There are no conflicts to declare.

## Supplementary Material

CP-025-D3CP01215H-s001
