# Supplementary material for: Adsorption dynamics of O2 on Cu(111): a supersonic molecular beam study
Source: Phys Chem Chem Phys. 2023 May 17;25(21):14862–8. doi: 10.1039/d3cp01215h (PMC10231304; doi:10.1039/d3cp01215h)
Supplement: CP-025-D3CP01215H-s001 [file CP-025-D3CP01215H-s001.pdf]

Electronic Supplementary Material (ESI) for Physical Chemistry Chemical Physics.
This journal is © the Owner Societies 2023

1 Supplemental information to Adsorption dynamics of O2 on Cu(111): a supersonic molecular beam study

Diyu Zhang,[a] Charlotte Jansen,[a] Aart W. Kleyn,[a] and Ludo B. F. Juurlink*[a]

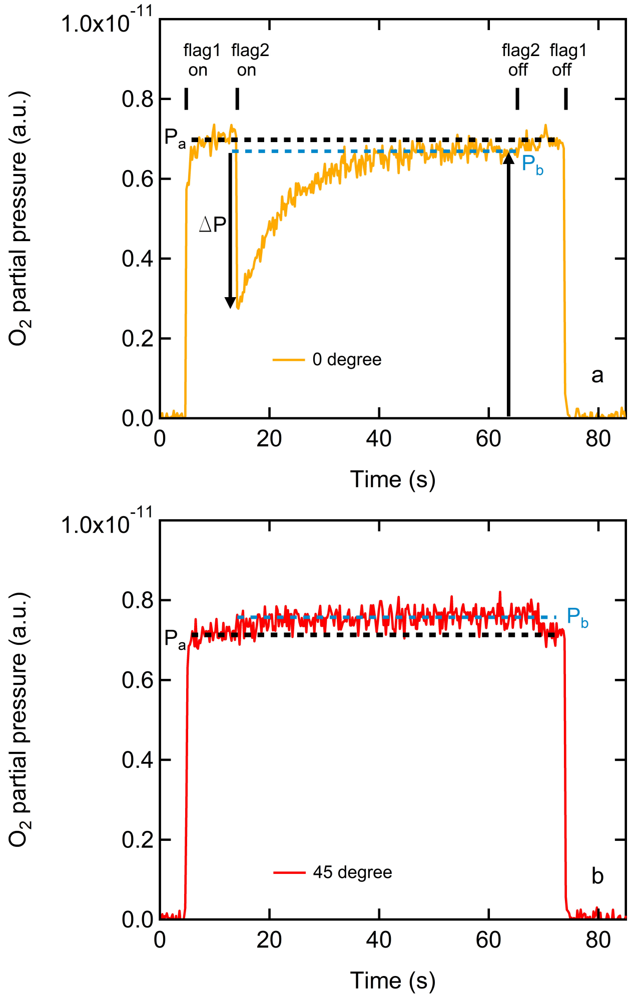

Figure S1 a sample of KW measurement of O_2 (6.5% in He) on Cu(111) at normal (a) and 45 degree (b) incident angle.

Figure S1a shows KW measurement of O_2 (6.5% in He) on Cu(111) at normal incident angle. KW measurement require two flags to modulate the molecular beam and a QMS to measure the partial pressure of the molecule that we are interested in. Both flags are initially closed. We first open flag 1 to have the molecular beam enter the UHV main chamber. It results in an O_2 partial pressure increase to the level indicated as P_a. At this time, the molecular beam is still blocked by flag 2, which prevents direct impact of O_2 onto the Cu(111) surface. We subsequently open flag 2, letting the molecular beam impinge directly onto the sample surface. The O_2 partial pressure drops immediately due to the O_2 (dissociative) adsorption. The O_2 pressure slowly increases again until the surface reaches its saturation coverage for oxygen. The O_2 partial pressure is stable at P_b. We then close flag 2 again. Although this should not have an effect as at saturation there should be no adsorption anymore, the partial pressure changes slightly from P_b back to P_a again. Finally, we close flag 1 and the absolute O_2 partial pressure drops back to the initial pressure.

The minor difference between P_b and P_a result from effective pumping speed variations resulting from the position of the turbo molecular pump and the QMS in comparison to the scattering surfaces. The molecular beam is highly directional. When it is blocked by flag 2 or scatters from the surface, the effective pumping speed is slightly different and we detect improved pumping speed resulting from scatting more toward the TMP's orifice as a lowered partial pressure. In other words, when the reflected beam is directed more toward the TMP, the pumping speed is higher, resulting in the lower detected O_2 partial pressure. When the reflected beam is, on the contrary, more directed toward the QMS,the detected O_2 partial pressure is higher. We show this by repeating the seeqeuential opening and closing of both flags, but change the incident angle to 45 degrees. Results are shown in the figure S1b. Now, no (dissociative) sticking is observed as the Cu(111) surface was truly saturated in the previous experiment, while more O_2 is now reflected toward the QMS. Now, P_b is also higher than P_a.

For technical reasons, repositioning the TMP and QMS to remove this effect of varying effective pumping speeds and measured partial pressures was not possible. Hence, the initial sticking probability (S_0) is calculated by

$$S_0 = \frac{\Delta P}{P_b} \quad (1)$$

where ΔP is the initial pressure drop as compared to the partial pressure level P_b, i.e. the ultimate pressure rise from the saturated Cu(111) surface at the position of the crystal scattering the entire beam. For our angle dependent measurements, we take the same effect into account in an identical sense.

[0a] *Leiden Institute of Chemistry, Leiden University, PO Box 9502, 2300 RA Leiden, The Netherlands Tel: (+31) 71 527 4221; E-mail: l.juurlink@chem.leidenuniv.nl*

Table S1 Average kinetic energy of O_2 at molecular beams with different seeding ratio

O_2 / He	average kinetic energy
0.014	396 meV
0.028	374 meV
0.070	332 meV
0.233	216 meV

[a] We notice that what the mixture actually is on the beam axis likely deviates somewhat from what is actually pushed into the gas line to the nozzle by the flow controllers.

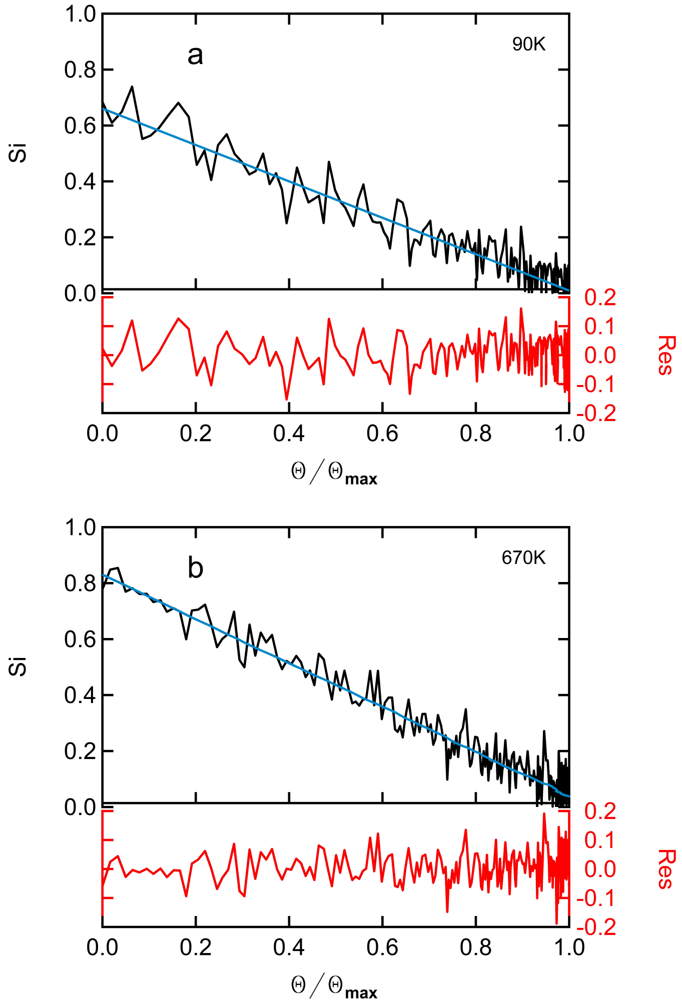

Figure S2 Linear fits to $S(\theta)$ as a function of θ/θ_{max} at 379 meV (normal incidence) for T_{surf} =90 K and 670 K.

Figure S2 shows two representative fits to $S(\theta)$ as a function of θ/θ_{max} at T_{surf} = 90 K and 670 K for the average kinetic energy of 379 meV. We fit the sticking data, S_i, shown in the top panels over the entire range with a linear function . We calculate the residuals between the data and the fit and plot these residuals in the bottom pane. If there was any significant deviation from linear behavior as suggested for N = 2 in the relative coverage dependence of S_i (as discussed in the main text), we should see curvature in these residuals with positive values at the start and end of the experiment. We find for all fits that the residuals fall into a straight band that likely mostly reflects the noise level of our QMS. Lack of any curvature indicates that $S(\theta)$ is strictly linear across the entire θ/θ_{max} range.
